# Cannula tip separation and configuration as determinants of recirculation in venovenous ECMO

**DOI:** 10.1051/ject/2026010

**Published:** 2026-09-15

**Authors:** Elliott T Worku, Michael G Pittard, Richard J Totaro

**Affiliations:** 1 Intensive Care Service, Royal Prince Alfred Hospital, Camperdown Sydney 2050 Australia; 2 Faculty of Medicine, University of Sydney Camperdown Sydney Australia

**Keywords:** Extracorporeal Membrane Oxygenation (ECMO), Acute Respiratory Distress Syndrome (ARDS), Recirculation, Cannula configuration

## Abstract

*Background*: Recirculation during venovenous extracorporeal membrane oxygenation (VV ECMO) is an important phenomenon that limits the efficiency of support. Femoro-jugular (FJ) cannulation may be associated with lower recirculation than femoro-femoral (FF) configurations. Local practice is to routinely overlap cannula in the FJ configuration; this approach remains poorly described, and its impact on recirculation is not understood. *Methods*: The saturation-based formula was used to assess recirculation fraction amongst 24 patients receiving VV ECMO between July 2018 and March 2025 in this single-centre retrospective study. All received VV ECMO via a multistage drainage cannula and a single-stage return. Factors considered relevant to recirculation were also collected at the time of a recirculation assessment, and cannula separation was obtained from chest X-ray imaging. Recirculation was compared between configurations, with further analysis where an overlap of cannula was present. Univariate and multivariate regression were performed to evaluate the influence on recirculation and clinical outcomes. *Results*: Demography was similar between configurations, with the majority of VV ECMO indications being ARDS secondary to bacterial pneumonia. Patients in whom recirculation was assessable were similar to those excluded. Recirculation was non significantly lower amongst patients with FJ cannulation and lacked a clear relationship to extracorporeal blood flow and drainage tip position. There were no significant differences in clinical outcomes between cannula configurations, numerically higher rates of tracheostomy and awake ECMO were observed for FJ cannulations. Older age and higher vasoactive burden were associated with length of stay and survival. *Conclusion*: There are a dearth of studies investigating recirculation in patients with deliberate overlap of femoral and jugular cannula, particularly in the context of large calibre multistage drainage devices. A prospective study is necessary to systematically evaluate the influence of this practice on VV ECMO efficiency, ideally using advanced recirculation measurements and contemporaneous cardiac output monitoring.

## Introduction

Veno-venous extracorporeal membrane oxygenation (VV ECMO) is indicated in the management of severe acute hypoxaemic and hypercapnic respiratory failure and is associated with survival benefit [1].

Some of that benefit is likely derived from reductions in mechanical power delivery to the native lung facilitated by extracorporeal gas exchange [2–5]. However, ultraprotective “rest” ventilation settings may worsen atelectasis [6, 7], while supraphysiological pulmonary arterial oxygen saturation may impair hypoxic pulmonary vasoconstriction [8, 9], exacerbating native lung shunt. Thus, high ECMO dependency is often incurred in the early post-implantation phase [10].

**Figure 1:**
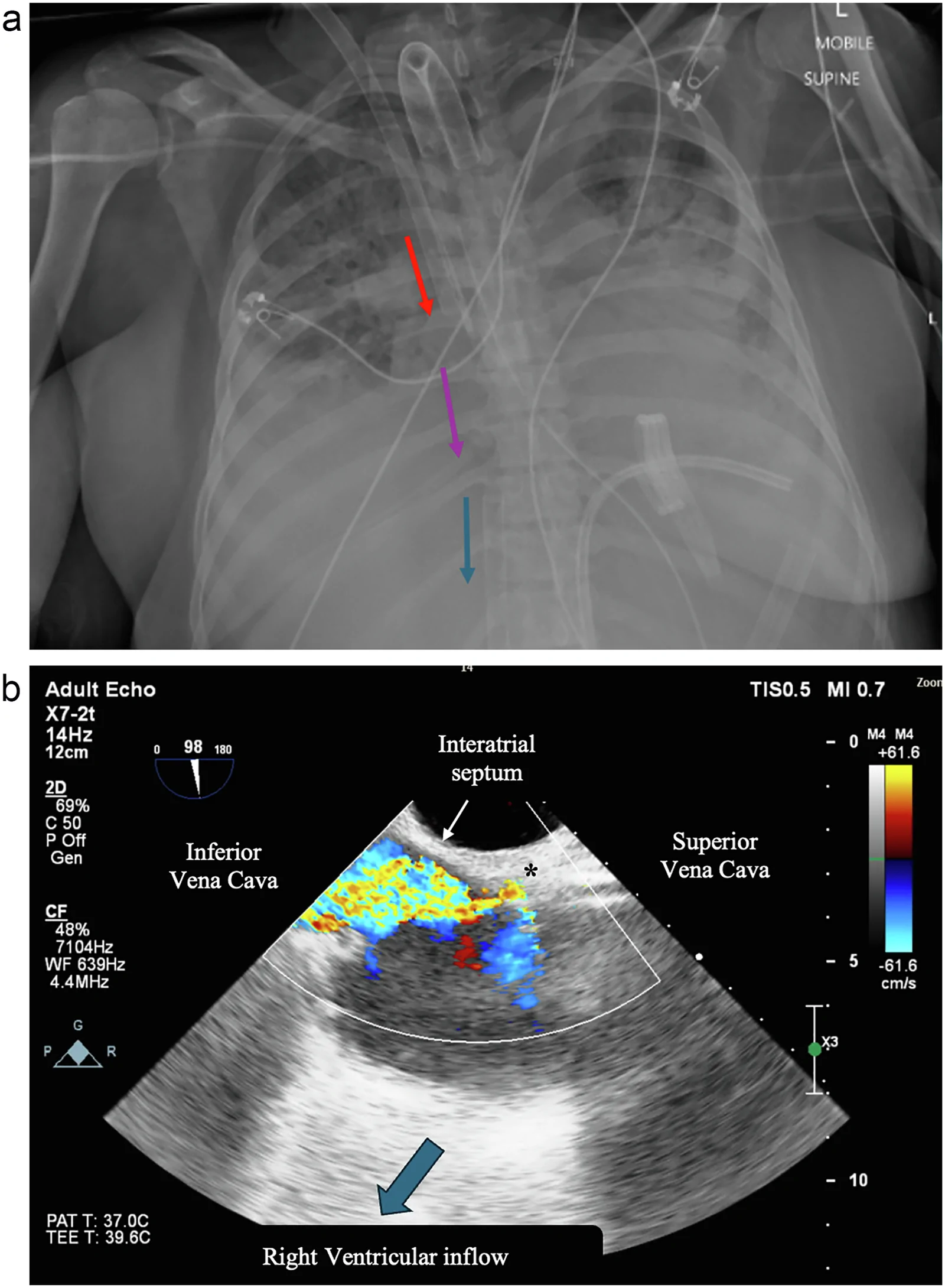
Plain film radiograph demonstrating a femoral multistage drainage cannula with tip in the IVC, and a right internal jugular vein single-stage return cannula with tip in the right atrium. Arrows illustrate flow direction and potential sites of recirculation: blue indicates drainage flow, red indicates return flow, and purple indicates recirculated blood re-entering the drainage cannula. IVC: Inferior Vena Cava.

The arterialised effluent exiting the ECMO membrane contains significantly elevated bound and solubilised oxygen [11], however, the portion that is delivered to the patient is influenced by recirculation. Recirculation describes the portion of return from the membrane oxygenator being entrained directly into the ECMO drainage cannula, rather than entering the systemic venous circulation [12] (Figure 1).

Increased recirculation may necessitate the imposition of therapies such as sedation, neuromuscular blockade, and fluid administration to achieve adequate oxygen delivery [13].

Persisting hypoxaemia due to recirculation may even require manipulation of cannula positioning, risking infective complications and accidental decannulation [14], or an additional cannula to facilitate high flow configurations [15]. Increasing extracorporeal blood flow (ECBF) can overcome the deleterious influence of recirculation [16], but at the cost of more negative venous access pressures, and potentially greater blood trauma.

Recirculation is a dynamic phenomenon influenced by patient and circuit factors [17, 18]. A dual lumen cannula anatomically separates caval drainage and reinfusion [12], inherently limiting recirculation, while multistage drainage devices are superior to single-stage drainage [12, 19, 20]. Computational fluid dynamics also suggests that within a multistage cannula, side hole design may differentially modulate shear stress and recirculation [21].

A higher ratio of cardiac output to ECBF reduces the recirculation fraction (Rf) [22, 23], however, this risks hypoxaemia on ECMO due to admixture [10]. Intrathoracic pressure [24], cannula tip separation and orientation of the return blood flow in relation to drainage may also be relevant [25, 26]. Of these, cannula positioning is a modifiable factor that clinicians can optimise.

Data comparing recirculation between femoro-jugular (FJ) and femoro-femoral (FF) configurations are inconsistent, the former may permit a larger drainage cannula to be used [27, 28]. Consistent clinical benefit to prefer one configuration over the other is lacking, and jugular instrumentation may entail higher procedural risk [29, 30].

**Figure 2:**
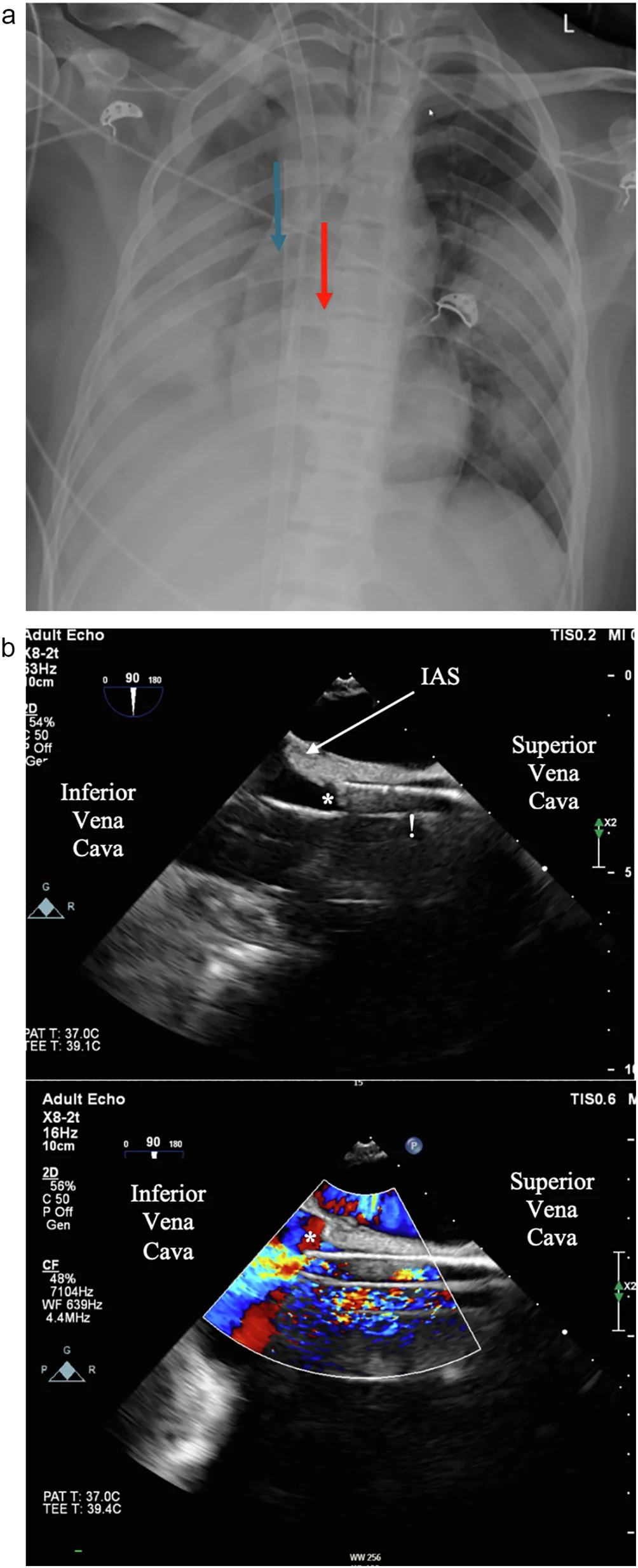
Plain film radiograph demonstrating a femoral multistage drainage cannula with tip in the SVC, and a right internal jugular vein single-stage return cannula with tip in the right atrium. Overlap and direction of inflow and return annotated with arrows: blue indicates drainage flow, and red indicates return flow. Key: SVC, Superior Vena Cava.

At our centre, femoro-jugular cannulation is performed in the majority of VV ECMO initiations (Supplementary Table 1). Uniquely, we intentionally target overlap of the femoral drainage and jugular return in our local practice (Figure 2).

We hypothesised that FJ cannulation would be associated with favourable recirculation and drainage haemodynamics compared to FF cannulation. Furthermore, we wished to describe recirculation characteristics in patients with this rarely reported overlap between drainage and return cannulas.

## Materials and methods

This retrospective monocentric study was conducted in accordance with the principles stated in the Declaration of Helsinki. The study protocol (X25-0188) was approved by the local ethics committee (2025/ETH01463), and the requirement for informed consent to participate was waived due to its observational and retrospective nature. Recirculation was measured according to the saturation-based equation described previously [17, 18].

$$\mathrm{Rf}\;\left(\%\right)=\left(S_{\mathrm{pre}}O_{2}–\mathrm{Sv}O_{2}\right)/\left(S_{\text{post}}O_{2}–\mathrm{Sv}O_{2}\right)\times 100$$

Pre; pre membrane, post; post membrane

Rf% = recirculation fraction (percentage)

### Patient population

Eligible patients were those receiving VV ECMO via two single-lumen cannulas in either femoro-femoral (FF) or femoro-jugular (FJ) configuration, from July 2018 (programme inception) until 1st March 2025. Patients receiving hybrid ECMO entailing an arterial return, a high flow configuration, or a dual lumen cannula at the time of a recirculation measurement were excluded.

### Recirculation measurements

Patients managed in our intensive care unit have clinical, physiological and ECMO data uploaded at hourly intervals to the electronic medical record (EMR) (Philips Intelli Space Critical Care and Anaesthesia, Philips Healthcare, The Netherlands). The EMR of each patient was reviewed to determine whether pre- and post-membrane blood gas analysis, and contemporaneous SvO_2_ measurements were available, permitting calculation of the recirculation fraction according to the CVL (central venous line) method and saturation equation at least once using the equation provided above [17]. Local practice is to reserve the femoral veins and right internal jugular for VV ECMO, and if necessary, escalation to high flow configuration, the left internal jugular vein is where the majority of 20 cm CVLs are placed, with the tip generally terminating in the SVC or right atrium. Daily pre- and post-membrane blood gases are not mandated in our service and thus were not present for all patients.

Simultaneous ventilator, physiological data, vasoactive inotropic scores [31], cumulative fluid balance and airway type were extracted. The CXR (chest X-ray) closest to the timing of a recirculation calculation was evaluated, and the cannula tip position and separation were assessed by an experienced ECMO specialist [32] In FJ cannulations, crossover of the access and return was denoted by a negative number (e.g., 2 cm crossover equates to −2 cm).

Outcome data were obtained from the EMR. Palliative decannulation with clinical documentation to that effect was deemed ECMO non-survival.

### Statistical analysis

Demographic and clinical characteristics were summarized using frequencies and percentages for categorical variables, and median (IQR) for continuous variables, given the small data set and assumption of non-normality. The Mann-Whitney U test was performed to compare the medians of categorical variables, and ANOVA to compare three or more groups. Correlations were assessed by Pearson’s coefficients, with significance set at a *P* value <0.05

To compare mortality outcomes, chi-squared analysis was performed using categorical data from each group. Multivariate linear regression was applied to factors previously identified to influence recirculation.

The primary outcome was the recirculation fraction based on configuration at the time of assessment and by the separation distance between drainage and return cannula as measured radiologically. We performed further analyses on various patient and ECMO-related factors, including drainage cannula size and drainage tip location [19]. Statistical analysis was performed using Microsoft Excel with the Analysis ToolPak add-in (Microsoft Corporation, Redmond, WA, USA).

## Results

**Figure 3:**
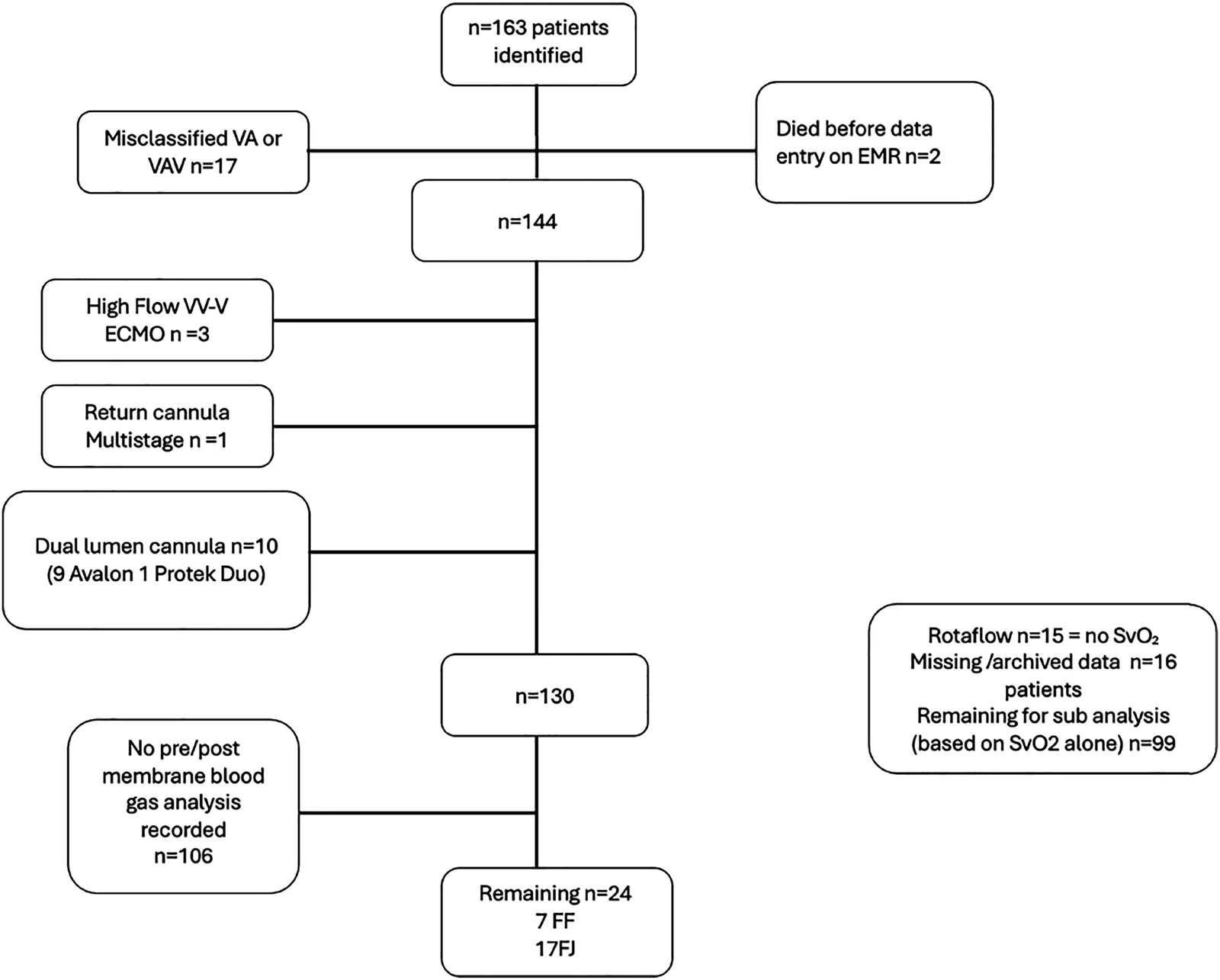
Box-and-whisker plot showing recirculation fraction (%) by cannulation configuration. Patients are grouped by femoro-femoral (FF) and femoro-jugular (FJ) venovenous ECMO configurations. There was no significant difference in recirculation fraction between groups. Key: FF – Femoro-femoral; FJ – Femoro-jugular.

During the study period, 163 patients were identified. After exclusions for misclassification of Veno arterial ECMO, hybrid configurations (VAV ECMO), dual lumen cannula, or for insufficient data (either lack of SvO_2_ or pre- and post-membrane blood gas analysis) to make a recirculation assessment, 24 patients remained (Figure 3). Of these, 17 underwent FJ, and 7 FF cannulations. Hybrid configurations excluded were high flow VV ECMO using two drainage cannula, and VAV (Veno arteriovenous) ECMO. In the former, there are two drainage cannula sharing the total ECBF, and no single cannula separation to report. In the latter, total circuit flow is diverted between an arterial and venous return; the arterial oxygen saturations and SvO_2_ are variably influenced by the arterial return and by the phenomenon of differential oxygenation, which would affect recirculation assessments in the VV portion of the circuit.

Of the 106 VV ECMO patients excluded from the primary study, 99 patients had SvO_2_ monitoring available. Using a SvO_2_ > 75% and hypoxaemia defined as SpO_2_ < 88%, we undertook to describe the demography, outcomes, recirculation incidence, and rates of recirculation-related hypoxaemia in these patients described in Supplementary Table 1.

Demography of the 24 patients in the main study is summarised in Table 1. There were no significant differences in patient age, sex, height, BMI, or ECMO indication between configurations. Overall, ARDS secondary to bacterial pneumonia was the most common aetiology, and COVID-19 was diagnosed in 20% (FJ) and 28.6% (FF) of patients, respectively (ns). Most patients were ventilated via an oral endotracheal tube during ECMO, with the majority receiving a pressure control mode (PCAC and PC SIMV); 50% of patients in the FJ group and 34.9% of the FF group (ns) were tracheostomised at the time of recirculation assessment.

**Table 1:** Patient demographics, diagnoses, and outcomes by cannulation configuration. Data are presented as median (IQR) or percentage as appropriate. Comparisons between groups were performed using the Mann–Whitney U test or Chi-square test (*).

**Demographics**	FJ (*n* = 17)	FF (*n* = 7)	*p*-value
Age	47 (40–56)	46 (40–52)	0.52
Sex (Male)	64.7%	71.4%	0.75
Height (cm)	170 (160–178)	173 (170–176)	0.70
BMI (Kg/m^2^)	31.3 (25.7–33.5)	24.2 (22.9–38)	0.13
**Diagnosis**			
Bacterial Pneumonia	41.2%	28.6%	0.75*
Viral Pneumonia	11.8%	14.3%	-
COVID	11.8%	28.6%	-
Aspiration	5.9%	14.3%	-
Misc	29.3%	14.3%	-
**Outcomes**			
ECMO duration (days)	11.2 (8.3–23.1)	29.2 (9.0–42.9)	0.18
ECMO survival	52.9%	55.5%	0.86
ICU LOS (days)	26 (16–41)	58 (28–84)	0.45
ICU Survival	52.9%	44.4%	0.32
Tracheostomy on ECMO	50%	34.9%	0.55*
Extubated on ECMO	33.3%	14.3%	0.26
Hospital LOS	48 (27–84)	87 (29–111)	0.45
Hospital Survival	52.9%	33.3%	0.34

BMI Body Mass Index; COVID-19 Coronavirus disease 2019; ECMO Extracorporeal Membrane Oxygenation; FF Femoro-femoral; FJ Femoro-jugular; ICU Intensive Care Unit; IQR Interquartile Range.

Awake ECMO (extubation before decannulation) was provided in one third of patients in the FJ group, and 14.3% in the FF group (ns).

There were no significant differences in ventilation and respiratory parameters, VIS, nor cumulative fluid balance; peripheral saturations were significantly lower in the FJ cohort, 93% vs. 99% (*p* = 0.05) (Table 2).

**Table 2:** Comparison of physiological and ECMO parameters at the time of recirculation measurement between femoro-jugular (FJ) and femoro-femoral (FF) cannulation configurations. Values are presented as median (interquartile range). Comparisons were made using the Mann–Whitney U test. † Effective flow calculated as: ECMO blood flow × (1 – Recirculation fraction/100).

	Femoro-Jugular cannulation	Femoro-Femoral cannulation	*p*-value
Cannula separation (cm)	5.7 (0.425–8.725)	5.5 (2.8–5.75)	0.63
ECBF (L/min)	4.45 (3.92–5.50)	4.18 (3.67–4.76)	0.30
Pump speed (RPM)	3003 (2674–3505)	3150 (2936–3240)	0.78
Access pressure (Pven, mmHg)	−57 (−80 to −44.5)	−43.5 (−64.25 to −34)	0.22
Recirculation fraction (%)	29.3 (8.5–48.7)	41.5 (27.3–47.6)	0.49
Effective flow (L/min)†	3.15 (2.48–3.58)	2.31 (1.52–4.47)	0.96
Sweep gas flow rate (L/min)	8 (5–9)	6 (2.5–7.38)	0.05
FdO2 (%)	100 (100–100)	100 (85–100)	0.43
Pre-membrane SO_2_ (%)	78 (69–83)	75 (71.3–77.8)	0.38
SvO_2_ (%)	63.5 (54–75.8)	56 (52–58.5)	0.19
Plasma free Hb (mg/dL)	640 (450–1090)	500 (240–990)	0.52
D-dimer (mg/L)	7.45 (2.28–19.32)	17.5 (3.93–18.72)	0.86
Hb (g/dL)	92 (78–101.5)	92 (82.3–103.5)	0.32
Cumulative fluid balance (mL)	3531 (−255–6509)	6564 (1941–8215)	0.2
Vasoactive-inotropic score	4.85 (0.25–18.93)	0.75 (0–6.95)	0.2
Peak airway pressure (cmH2O)	20 (23–26)	23.5 (21.3–25.5)	0.76
PEEP (cmH_2_O)	12 (10–12)	10 (9–11)	0.23
Respiratory rate (bpm)	12 (10–15.5)	10 (10–21)	0.83
Expired tidal volume (mL)	220 (66–384)	177 (139–287)	0.91
FiO_2_ (%)	60 (60–77.5)	60 (50–60)	0.11
SaO_2_ (%)	93 (87.8–96.3)	99 (95.3–99.8)	0.05
PaO_2_ (mmHg)	66 (58–86)	94.5 (75.3–106.3)	0.15
PaCO_2_ (mmHg)	43 (37.5–53.5)	42.5 (39–47.5)	0.76
PaO_2_ FiO_2_ ratio	101 (79–147.5)	193.5 (113–222.3)	0.16

ECBF: Extracorporeal blood flow; ECMO: Extracorporeal membrane oxygenation; FdO_2_: Fraction of delivered oxygen; FF: Femoro-femoral; FiO_2_:Fraction of inspired oxygen; FJ: Femoro-jugular; Hb: Haemoglobin; PaCO_2_: Arterial partial pressure of carbon dioxide; PaO_2_: Arterial partial pressure of oxygen; PEEP: Positive end-expiratory pressure; Pven: Access pressure; SaO_2_: Arterial oxygen saturation; SO_2_: Oxygen saturation; SvO_2_: Venous oxygen saturation (measured on ECMO console); VIS: Vasoactive-inotropic score; RPM: Revolutions per minute.

### Recirculation fraction

**Figure 4:**
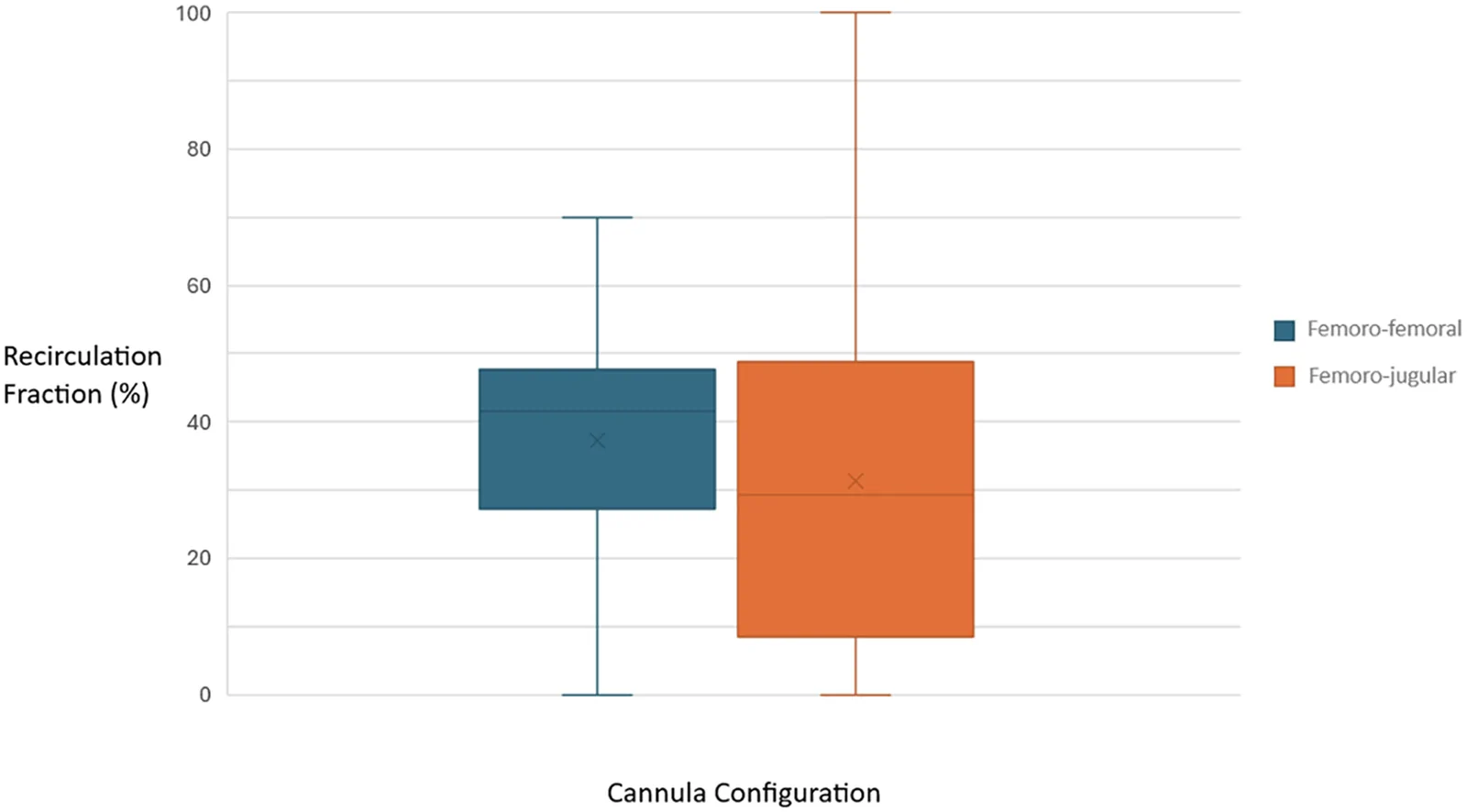
Recirculation fraction (%) stratified by access cannula tip position in femoro-femoral (FF) and femoro-jugular (FJ) VV ECMO configurations. Tip position determined by chest X-ray and categorised as superior vena cava (SVC), right atrium (RA), or inferior vena cava (IVC) [19]. There was no significant difference in recirculation fraction between groups. Key: FF: Femoro-femoral; FJ: Femoro-jugular; NS: non significant; SVC: Superior vena cava; RA: Right atrium; IVC: Inferior vena cava; VV ECMO: veno-venous extracorporeal membrane oxygenation.

The median recirculation fraction was non significantly lower in FJ compared with FF cannulations, 29.3% (8.5; 48.7) vs. 41.5 (27.3; 47.6) *p* = 0.49 (Figure 4).

There was no difference in SvO_2_ between FF and FJ [56% (52; 58.5) vs. 63.5% (54; −75.8), *p* > 0.1] (Table 2).

### ECMO characteristics

Extracorporeal blood flow was similar (4.45 vs. 4.18 LPM, *p* = 0.3), as were pump speed, venous access pressure, and FdO_2_. The effective ECMO blood flow [ECBF × (1 − Rf/100)] was non significantly higher in FJ [3.15 (2.48; 3.58) vs. 2.31 (1.52; 4.47) *p* = 0.96] cannulations. Sweep gas flow rate was higher in patients with FJ cannulation (8 vs. 6 LPM, *p* = 0.05) (Table 2).

### Cannula size

Similar multistage drainage cannula were used between configurations (Supplementary Table 2). In FF cannulations, 21Fr long single-stage return cannulas (Maquet, Getinge, Rastat, Germany) were used exclusively, but a range of short “arterial cannula” from 19–23Fr were employed in FJ cannulations. Both Maquet (Getinge, Rastatt, Germany) and Bio-medicus (Medtronic, Dublin, Ireland) drainage and return cannula were used. When Rf was compared by drainage and return cannula size, no differences emerged (Supplementary Table 3).

### Cannula separation

Median cannula separation was similar between FJ and FF cannulation: 5.7 (0.4; 8.7) vs. 5.5 (2.8; 5.8) cm, *p* = 0.63. Within the FJ group, significant differences emerged; 7.0 (3.9; 9.0) cm vs. −2.3 (−3.2; −1.9) cm, *p* < 0.001, and between all three groups (FJ crossed, FJ uncrossed, FF) *p* < 0.001 (Table 3).

**Table 3:** Comparison of physiologic and circuit variables between femoro-jugular (FJ) ECMO patients with crossed vs uncrossed cannulation, and femoro-femoral (FF) patients. Data are presented as median (interquartile range). P values represent pairwise comparison between crossed and uncrossed FJ groups (Mann–Whitney U test), and between all three groups using one-way ANOVA.

	FJ Crossed	FJ Uncrossed	*p*-value (FJ groups)	FF	*p*-value (ANOVA)
Cannula separation (cm)	−2.3 (−3.2 – −1.9)	7.0 (3.9–9.0)	<0.0001	5.5 (2.8–5.8)	<0.00001
Recirculation fraction (%)	30.2 (6.8–46.5)	29.4 (11.4–46.2)	0.83	41.5 (27.3–47.6)	0.79
ECBF (L/min)	4.32 (3.66–4.89)	4.45 (3.62–5.52)	0.48	4.18 (3.67–4.76)	0.50
Effective flow (L/min)	2.51 (1.91–3.57)	3.19 (2.65–3.57)	0.52	2.31 (1.52–4.47)	0.90
Pump speed (RPM)	2975 (2660–3315)	3030 (2715–3540)	0.77	3150 (2936–3240)	0.89
Venous pressure (mmHg)	−36.5 (−27.0 – −57.3)	−61.0 (−50.0 – −80.0)	0.69	−43.5 (−64.3 – −34.0)	0.06
Pre-membrane saturation (%)	82 (77–84)	76 (68–80)	0.37	75 (71–78)	0.66
PEEP (cmH_2_O)	10 (8–14)	12 (10–12)	0.87	10 (9–11)	0.45
Respiratory rate (bpm)	11 (10–15)	12 (10–14.5)	0.98	10 (10–21)	0.88
Mean airway pressure (Paw, cmH_2_O)	20 (20–28)	23.5 (20–26)	0.76	23.5 (21.3–25.5)	0.81
Expired tidal volume (Vte, mL)	231 (220–320)	219 (55–401)	0.76	177 (139–287)	0.95
VIS	5.75 (0–25.95)	4.85 (1.75–13.25)	0.83	0.75 (0–6.95)	0.48
Cumulative fluid balance (mL)	4716 (3302–6249)	1796 (−488–7096)	0.45	6564 (1941–8215)	0.17

Key: ECBF: Extracorporeal blood flow; FJ: Femoro-jugular; FF: Femoro-femoral; PEEP: Positive end-expiratory pressure; RPM: Revolutions per minute.

Significant correlation was not found between Rf and cannula separation overall.

In the FJ group, there was a moderate statistically significant correlation in the uncrossed configuration (*r* = 0.48, *p* = 0.03).

A simple quadratic polynomial regression was applied for FJ cannulations, revealing a U-shaped line of best fit with weak, non-significant correlation (*r*^2^ = 0.21, *p* = 0.07).

### High and low separation

Recirculation fraction was assessed above and below 5 cm of cannula separation using unpaired t tests. This value was recently demonstrated to be an inflection point for recirculation in a bench study of ECMO using the ultrasound dilution method [22].

Recirculation was non significantly higher when FF cannula separation was less than 5 cm (Supplementary Figure 1). Recirculation trended to be higher when FJ cannulas were more than 5 cm apart (ns). In the case of crossed cannulas, we chose to explore Rf above and below the median overlap distance of 2.5 cm to permit more equitable subgroups (Supplementary Figure 2). Mean recirculation fraction was significantly higher when the cannulas had greater overlap; 53.0% (2.83) vs. 15.1% (17.6), *p* < 0.01.

These trends align with the possibility of a U-shaped relationship teased previously.

### Cannula sub-configurations

**Figure 5:**
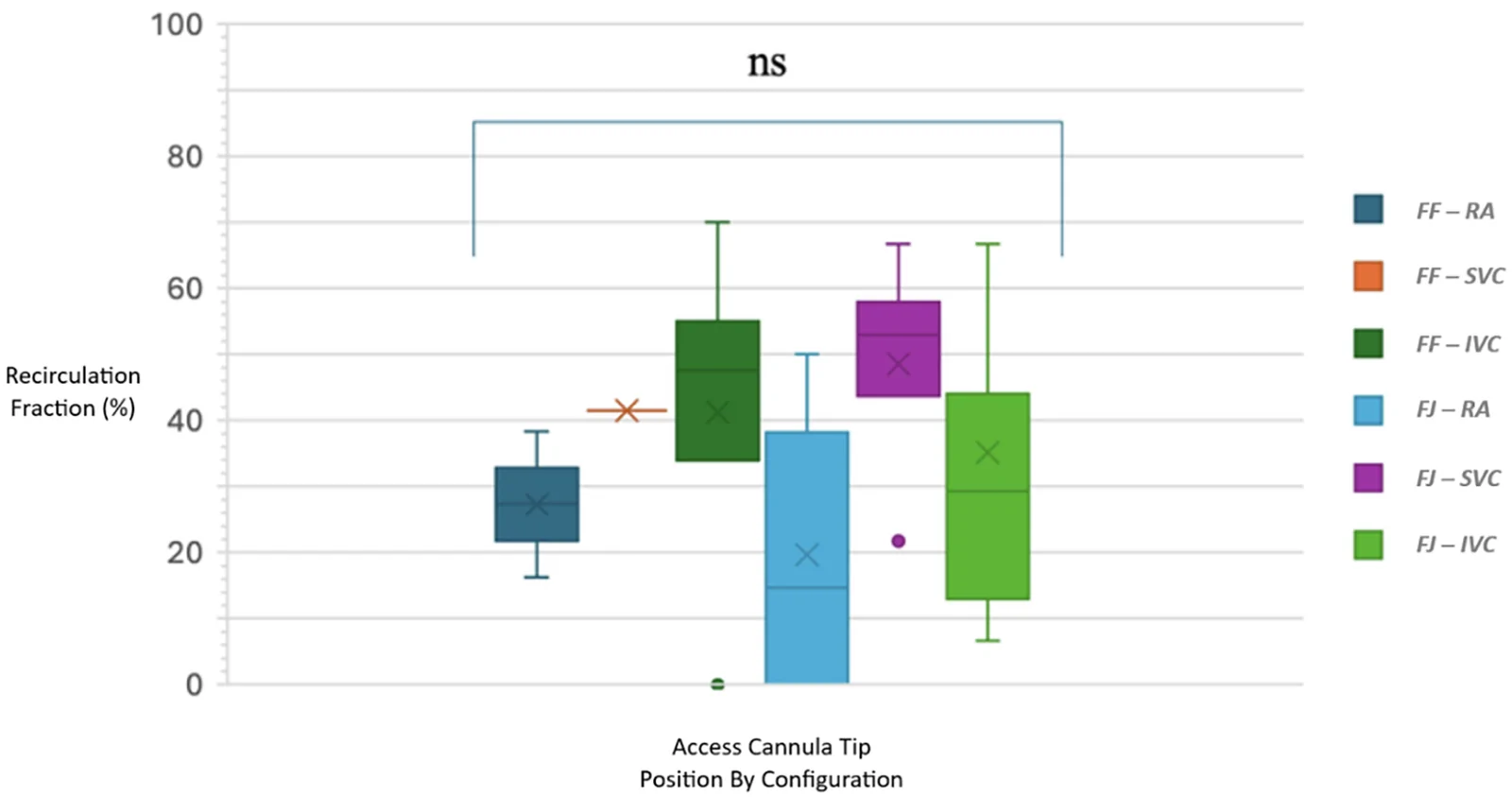
Configuration of access cannula tip position vs. recirculation fraction.

We further divided patients into 6 subgroups based on configuration and drainage tip position [19] (Figure 5). There were no significant differences in Rf between all six groups.

### ECBF and recirculation

Strong negative correlation between ECBF and Rf was found for FF cannulations, *r* = 0.881, *p* = 0.02. No relationship was evident for FJ cannulation, nor for the cohort as a whole (*r* = −0.09, *p* = 0.63).

### Flow dynamics

Flow was significantly higher however when a 29Fr drainage cannula was used instead of a 25Fr cannula; 1.50 (1.43; 1.58) vs. 1.34 (1.25; 1.42) Litres per 1000 RPM, *p* < 0.01.

Weak positive correlation was evidenced between cannula separation and ECBF/1000 RPM, *r* = 0.3125, *p* < 0.05.

### Clinical outcomes

ECMO duration, ICU LOS, and hospital LOS were all non-significantly shorter in patients who underwent FJ cannulation (Table 1). There was no missing data. Survival did not differ at any time point. One patient in the FJ cohort proceeded to organ donation on ECMO, and one patient in the FF group was transferred to an external centre for consideration of lung transplantation. Despite a lengthy hospital episode following transplant, this patient died. As both ECMO duration and ICU LOS were influenced by the wait for organ allocation, analyses were repeated with this outlier patient excluded. This did not significantly alter results.

### Univariate analysis

Pearson’s correlation coefficients were assessed for cumulative fluid balance (*r* < 0.05), peak airway pressure (*r* = −0.28, *p* = 0.25), tidal volume (*r* = −0.21, *p* = 0.2), PEEP (*r* = −0.18, *p* = 0.4), drainage cannula size (*r* = −0.156, *p* = 0.39), and return cannula size (*r* = 0.082, *p* = 0.65), with no significant results elicited.

### Multivariate regression

Regression was performed to evaluate recirculation fraction in models combining configuration, cannula separation, ECBF, Pven, VIS, CFB, and PEEP, with no significant results yielded. Multivariate analysis was subsequently performed for clinical outcomes based on age, BMI, gender, configuration, Rf, VIS, and cumulative fluid balance. Only age and VIS produced significant results (Table 4).

**Table 4:** Multivariate linear regression analysis of age and vasoactive-inotropic score (VIS) in relation to key clinical outcomes. Values are presented as regression coefficients (standard error), with associated p-values. Non-significant results are denoted as ns. Models were adjusted for body mass index (BMI), gender, cannulation configuration, recirculation fraction (Rf), and cumulative fluid balance (CFB).

Predictor	Outcome	Beta coefficient (SE)	*p*-value
Age	ECMO Duration	–0.442 (0.217)	0.057
	ICU LOS	–1.044 (0.326)	<0.01
	Hospital LOS	–1.987 (0.610)	<0.01
VIS	ECMO Duration	ns	ns
	ICU LOS	–0.830 (0.269)	<0.01
	Hospital LOS	–1.666 (0.504)	<0.01

Key: VIS: Vasoactive-inotropic score; LOS: Length of stay; ECMO: Extracorporeal membrane oxygenation; Rf: Recirculation fraction; CFB: Cumulative fluid balance.

### Secondary cohort

Ninety-nine patients ineligible for the main study were analysed separately (Supplementary Table 1). Their SvO_2_ values were transcribed from the ECMO console (Cardiohelp^®^, Getinge, Rastatt, Germany). Recirculation was defined as SvO_2_ > 75%, sustained if present for >4 h; recirculation-related hypoxaemia was defined as SpO_2_ < 88% occurring during a period of high SvO_2_ or documented circuit intervention explicitly performed to ameliorate recirculation.

Of the 99 patients, the majority (82.8%) underwent FJ cannulation, of which 63.3% were uncrossed. Overall, 60% of patients were male, higher in the FJ uncrossed cohort (76.9% uncrossed vs 40% crossed). FF patients were cannulated with a 25Fr multistage drainage cannula, in FJ cannulations, a 29Fr drainage cannula predominated, and both groups received a 21Fr single-stage return cannula. The incidence of “recirculation” was 50.5%, and sustained high SvO_2_ was demonstrated in 32.3% – non different between groups. Hypoxaemia associated with recirculation occurred in 7.1% of the 99 ECMO runs, and was sustained in 5.1%, again not different between groups. Ten patients in total underwent interventions to manage recirculation-related hypoxaemia: 9 cannula retractions, 1 upgrade to high flow VV ECMO. Clinical outcomes did not differ between configurations, ECMO duration was 8 days, ECMO survival 70.7%, and 25.3% of patients underwent tracheostomy. Intensive care survival was 53.5% overall. Intensive care unit survival was essentially the same as that observed in the main study cohort. However, the 24 patients with recirculation assessments in the main study cohort experienced longer ECMO durations, particularly for the FF group (median 29 days), although this was not assessed for statistical significance. Overall, the recirculation cohort of 24 patients included in the main study appears reasonably representative of the larger VV ECMO cohort managed at our centre during the study period.

## Discussion

Recirculation is an important and prevalent phenomenon during VV ECMO, influenced by both the ECMO circuit and patient-related factors, including cannula design, configuration, and cardiac output, body position, and ECBF [17, 18]. High levels of recirculation may have implications for hypoxaemia during ECMO, and be deleterious to aspects of patient care such as fluid balance management [33–35], and awake ECMO [36]. Cannula geometry represents a modifiable and important determinant of VV ECMO efficiency.

Our study failed to identify significant differences in recirculation fraction by configuration, but this was numerically lower in FJ cannulations [27, 37] and the values we obtained broadly agree with those reported elsewhere [19].

Fisser echoed a similar pattern of results in a bicentric study and also found a correlation between Rf and cannula separation [38]. In this study, the Regensberg patients received a short 21Fr (38 cm) femoral access cannula (implying IVC drainage), with jugular return. In contrast the Karolinska cohort received larger 25Fr drainage via the jugular vein, advanced to sit in the more capacious right atrium. In both centres, cannula separation was substantial, which, in conjunction with relatively low ECBF, justifies the very low recirculation values yielded in this study [38].

In our experience, the relationship between cannula separation and recirculation was not as clear. We found greater recirculation when femoral drainage cannula were more proximate (< 5 cm apart) [22], but in the FJ configuration, the relationship was more complex, possibly representing a U-shaped relationship.

When cannula tips are distracted, the drainage inflow may align directly with the pressurised return blood (Figure 1). However, when the tips are adjacent, this direct confrontation is minimised. Once the cannulas overlap, there may be an increase in recirculation by entrainment via the side holes of the multistage drainage cannula (Figure 2) [26]. When the tips were adjacent rather than separated or crossed, this generally pertained to the cannula tips both residing in the right atrium. In patients with high CO to ECBF ratios, this could prefer forward flow of arterialised return through the tricuspid valve, reducing recirculation [23]. However, this may be highly dependent on individual right atrial/vena cava haemodynamics [39], heart rate [23], and precise orientation of the return cannula to the tricuspid valve [16, 39] Given that a significant proportion of the inflow for the multistage cannula is achieved through the side holes, positioning of the drainage cannula is likely crucial [12, 16], as is the ratio of SVC to IVC blood flow [39].

Despite our preference for overlap of the ECMO cannula in the FJ configuration, this situation is only anecdotally represented in the literature [19, 26, 40, 41].

In one case report, dynamic assessment of recirculation using the ultrasound dilution method was employed to optimise drainage cannula positioning, resulting in a crossed cannula configuration [26]. Modifications to the return cannula mandrel have also been described, such that the return jet is steered towards the tricuspid valve. This adjustment has been associated with favourable recirculation characteristics and improvements in patient oxygenation [40, 41] In a computational fluid model, altering lie of the return cannula from 0° to 30° was associated with a reduction in recirculation fraction from approximately 30% to less than 10% [39], reinforcing the importance of return cannula orientation in relation to right ventricular inflow.

We found ECBF to be strongly negatively correlated with Rf in FF cannulations, but not in FJ cannulations, whereas a positive relationship is often reported [23, 38]. This may be due to the influence of omitted variables, including native cardiac output. Despite higher recirculation fractions with increased ECBF, Gehron et al. found that higher ECBF was also associated with linear increases in membrane oxygen transfer and subsequently peripheral oxygen saturations [23]. In a bench study, the ratio of cardiac output to ECBF was negatively correlated with Rf, and at CO/ECBF ratios >1.5, single lumen cannula sizes exerted minimal effect on modelled recirculation [22].

While we used larger drainage and return cannulas than generally reported [19, 38], cannula size did not influence Rf.

Larger drainage size did, however, afford more blood flow for a given pump speed; in this way, larger multistage cannulas may support sufficient ECBF and avoid the need for hybrid reconfigurations to manage hypoxaemia [23].

Jung et al. recently described similar Rf between FF and FJ cannulations measured using the ultrasound dilution method [19] The authors subdivided cannulations by drainage tip location, failing to elicit a correlation between recirculation and cannula separation, nor by drainage tip position. The authors surmised that the influence of configuration on recirculation may be limited when such multistage devices are implanted [19].

We similarly failed to find differences in recirculation by drainage tip location (Figure 5), which again may relate to the size and design of our drainage cannula. One study reported greater transition to awake ECMO and more negative fluid balance in patients with a drainage tip in the atrium as opposed to the IVC, suggesting auxiliary benefits to consider [34].

Significant recirculation lacks a consensus definition, but an arbitrary threshold of Rf > 30–35% has been suggested [23]. Pragmatically, any degree of recirculation that necessitates patient or circuit intervention to ameliorate hypoxaemia should be considered relevant. Peripheral saturations less than 90% (nadir 85%) were measured in approximately one quarter of our cases, with one third of ventilator FiO_2_ set >60% (70–100%) suggesting that recirculation may have been significant, however hypoxaemia during VV ECMO may be multifactorial, relating to the interaction between ECBF and native CO, and native consumption and thus cannot be attributed in retrospect solely to recirculation. However, no patients underwent remedial cannula manipulations specifically to address recirculation according to the EMR.

We observed a non-significant trend towards higher tracheostomy (50% vs. 34.9%), and higher rates of extubation before ECMO decannulation in the FJ cohort (33.3% vs. 14.3%). Coupled with numerically lower cumulative fluid balance and the trend in clinical outcomes for the FJ patients, there is compelling indication to repeat evaluation in a prospective manner.

Advanced age and higher vasoactive inotropic score at the point of recirculation measurements were the only factors associated with clinical outcomes, with greater shock associated with longer durations of care and poorer survival.

## Limitations

While our study is subject to the caveats of retrospective unicentric design, the dearth of data points and limited individual patient measurements reflects the infrequency with which objective recirculation assessments are made within our high-volume service. The requirement for non-mandatory blood tests to enable recirculation assessment potentially introduces sampling bias. It remains unknown to what extent recirculation was present, and its ramifications in excluded patients. Severe hypoxaemia during VVECMO is rare within our cohort; typically, just 1 or 2 VV ECMO patients undergo circuit modifications such as high flow reconfiguration per annum. Discrete assessments of recirculation based on single timepoints during ECMO runs (median 11 days FJ and 29 days FF) may not provide meaningful insights with respect to the degree of recirculation, nor the scope of impact on patients and outcomes. We did not indicate where, in each individual ECMO journey, recirculation was measured. Episodes of high Rf may be relevant at initiation and periods of high ECMO dependency, but less so when approaching liberation.

The lack of a significant effect in recirculation by configuration may be a result of low power or due to the proximity of cannulas in the femoro-jugular configuration, obviating potential differences. In the bench study by Bukova et al., high recirculation occurred with less than 5 cm separation. At this proximity, recirculation was independent of factors such as cardiac output and ECBF [22].

Although all patients underwent initial echocardiography, acute cor pulmonale is prevalent in ARDS patients [42], and may have developed later, potentially impacting recirculation [17, 43].

We were compelled to use the CVL method to assess recirculation, which, although well described, may not accurately represent mixed venous saturations in both the SVC and IVC, depending on the sampling lumen position [12, 18]. The veracity of SvO_2_ measurements can be enhanced by cessation of sweep gas [44], and by an oxygen content-based approach [45]. However, interruption to ECMO support, even brief, may be associated with significant patient decompensation, and tolerance of SGOT may indicate readiness for liberation when recirculation has less deleterious potential [46].

The ultrasound dilution method has emerged as a non-invasive technique permitting repeated recirculation measurements [17, 19, 23, 37, 44, 47]. It entails administration of a small volume of saline to the post membrane circuit; the ensuing haemodilution alters the measured ultrasound velocity. By comparing the areas under the curve for Doppler velocities in the pre- and post-membrane circuit, a ratio is produced corresponding to the degree of recirculation [17]. The increasing utilisation of this method may provide a standardisation of recirculation assessment, and use has been demonstrated in situations involving cannula overlap [26]. Unfortunately, this device is not licensed locally.

Thermal and indicator dilution-based methods of recirculation assessment also exist [17], with the added advantages of simultaneous cardiac output measurement [48].

Our study was also not powered to evaluate haemolysis, which did not differ between configurations [38]. Both Rotaflow^®^ (Getinge, Rastatt, Germany) and Cardiohelp^®^ (Getinge, Rastatt, Germany) ECMO consoles were used at our institution, which have been shown to have differing haemodynamic performance [49], however, data could not be analysed by the platform. Whether the cannula size [39, 50] and tip positioning modulates blood trauma [38] is similarly beyond scope.

Finally, cannula separation was assessed from plain film chest radiographs by an accredited ECMO practitioner. The inherent limitations of this modality mean that actual separation in 3D space may be overestimated or underestimated [51]. Fisser et al. identified different strengths of association for the relationship between cannula separation and recirculation depending on the imaging modality from which separation was measured [38]. While our method for measurement was internally consistent, CT imaging offers better appreciation of the orientation of cannula to one another, cava and right atrium, and the orientation of the return cannula with respect to the tricuspid valve.

## Conclusion

There is an evident need to more comprehensively measure and define clinically relevant recirculation, and to further characterise this phenomenon in cases of cannula overlap. As such, intentional cannula overlap in the femoro-jugular configuration cannot be recommended currently.

Recirculation is dynamic and requires longitudinal evaluation to understand its clinical impact on ECMO efficiency and clinical outcomes.

Future studies should include repeated measures of recirculation, including during awake ECMO and ambulation.

## Data Availability

All available data are incorporated into the article.

## Supplementary material

*Supplementary Figure 1*: Box-and-whisker plot showing recirculation fraction (%) in patients with femoro-femoral venovenous ECMO, stratified by drainage-to-return cannula tip separation on chest radiograph. Patients are grouped by separation distance <5 cm or ≥5 cm as assessed by an experienced ECMO specialist. No significant difference in recirculation fraction was observed between groups. *Supplementary Figure 2*: Box-and-whisker plots showing recirculation fraction (%) in femoro-jugular venovenous ECMO patients, stratified by cannula tip separation on chest radiograph. Top panel: Patients with uncrossed cannulas, grouped by separation <5 cm vs ≥5 cm. No statistically significant difference. Bottom panel: Patients with crossed cannulas, grouped by overlap <2.5 cm vs ≥2.5 cm. Recirculation fraction was significantly lower in patients with greater tip separation (*p* < 0.01). *Supplementary Table 1*: Assessment of potential recirculation in 99 patients excluded from the primary study based on SvO_2_ monitoring. *Supplementary Table 2*: Cannula sizes used by configuration. *Supplementary Table 3*: Recirculation by Drainage and Return Cannula Size in FJ and FF Configurations. The supplementary material of this article is available at https://ject.edpsciences.org/10.1051/ject/2026010/olm.

## References

[1] Goligher EC, Tomlinson G, Hajage D, Wijeysundera DN, Fan E, Juni P, et al. Extracorporeal membrane oxygenation for severe acute respiratory distress syndrome and posterior probability of mortality benefit in a post hoc Bayesian analysis of a randomized clinical trial. JAMA. 2018;320(21):2251–2259. 10.1001/jama.2018.14276. 30347031

[2] Spinelli E, Carlesso E, Mauri T. Extracorporeal support to achieve lung-protective and diaphragm-protective ventilation. Curr Opin Crit Care. 2020;26(1):66–72. 10.1097/MCC.0000000000000686. 31876625

[3] Serpa Neto A, Deliberato RO, Johnson AEW, Bos LD, Amorim P, Pereira SM, et al. Mechanical power of ventilation is associated with mortality in critically ill patients: an analysis of patients in two observational cohorts. Intensive Care Med. 2018;44(11):1914–1922. 10.1007/s00134-018-5375-6. 30291378

[4] Schmidt M, Pham T, Arcadipane A, Agerstrand C, Ohshimo S, Pellegrino V, et al. Mechanical ventilation management during extracorporeal membrane oxygenation for acute respiratory distress syndrome. An International Multicenter Prospective Cohort. Am J Respir Crit Care Med. 2019;200(8):1002–1012. 10.1164/rccm.201806-1094OC. 31144997

[5] Cressoni M, Gotti M, Chiurazzi C, Massari D, Algieri I, Amini M, et al. Mechanical power and development of ventilator-induced lung injury. Anesthesiology. 2016;124(5):1100–1108. 10.1097/ALN.0000000000001056. 26872367

[6] Worku ET, Yeung F, Anstey C, Shekar K. The impact of reduction in intensity of mechanical ventilation upon venovenous ECMO initiation on radiographically assessed lung edema scores: A retrospective observational study. Front Med (Lausanne). 2022;9:1005192. 10.3389/fmed.2022.1005192. 36203770 PMC9531725

[7] Jamadar DA, Kazerooni EA, Cascade PN, Fazzalari FL, Vydareny KH, Bartlett RH. Extracorporeal membrane oxygenation in adults: radiographic findings and correlation of lung opacity with patient mortality. Radiology. 1996;198(3):693–698. 10.1148/radiology.198.3.8628856. 8628856

[8] Reis Miranda D, van Thiel R, Brodie D, Bakker J. Right ventricular unloading after initiation of venovenous extracorporeal membrane oxygenation. Am J Respir Crit Care Med. 2015;191(3):346–348. 10.1164/rccm.201408-1404LE. 25635492

[9] Mendes PV, Park M, de Azevedo LCP, Morais CCA, Amato MBP, Costa ELV. Lung perfusion during veno-venous extracorporeal membrane oxygenation in a model of hypoxemic respiratory failure. Intensive Care Med Exp. 2022;10(1):15. 10.1186/s40635-022-00442-x. 35467225 PMC9038965

[10] Schmidt M, Tachon G, Devilliers C, Muller G, Hekimian G, Brechot N, et al. Blood oxygenation and decarboxylation determinants during venovenous ECMO for respiratory failure in adults. Intensive Care Med. 2013;39(5):838–846. 10.1007/s00134-012-2785-8. 23291732

[11] Muller MC, Wilke SK, Dobbermann A, Kirsten S, Russ M, Weber-Carstens S, et al. Dissolved oxygen relevantly contributes to systemic oxygenation during venovenous extracorporeal membrane oxygenation support. ASAIO J. 2024;70(8):667–674. 10.1097/MAT.0000000000002171. 38446867 PMC11280439

[12] Palmer O, Palmer K, Hultman J, Broman M. Cannula design and recirculation during venovenous extracorporeal membrane oxygenation. ASAIO J. 2016;62(6):737–742. 10.1097/MAT.0000000000000440. 27660904 PMC5098462

[13] Nunes LB, Mendes PV, Hirota AS, Barbosa EV, Maciel AT, Schettino GP, et al. Severe hypoxemia during veno-venous extracorporeal membrane oxygenation: exploring the limits of extracorporeal respiratory support. Clinics (Sao Paulo). 2014;69(3):173–178. 10.6061/clinics/2014(03)05. 24626942 PMC3935134

[14] Crnich CJ, Maki DG. The promise of novel technology for the prevention of intravascular device-related bloodstream infection. II. Long-term devices. Clin Infect Dis. 2002;34(10):1362–1368. 10.1086/340105. 11981732

[15] Dave SB, Leiendecker E, Creel-Bulos C, Miller CF, Boorman DW, Javidfar J, et al. Outcomes following additional drainage during veno-venous extracorporeal membrane oxygenation: A single-center retrospective study. Perfusion. 2025;40(3):647–656. 10.1177/02676591241249609. 38756070

[16] Leoni M, Szasz J, Meier J, Gerardo-Giorda L. Blood flow but not cannula positioning influences the efficacy of Veno-Venous ECMO therapy. Sci Rep. 2022;12(1):20950. 10.1038/s41598-022-23159-z. 36470881 PMC9722702

[17] Gagliardi V, Gagliardi G. Recirculation in veno-venous extracorporeal membrane oxygenation. Medicina (Kaunas). 2024;60(12). 10.3390/medicina60121936. PMC11676060 39768819

[18] Xie A, Yan TD, Forrest P. Recirculation in venovenous extracorporeal membrane oxygenation. J Crit Care. 2016;36:107–110. 10.1016/j.jcrc.2016.05.027. 27546757

[19] Jung YC, Chong Y, Han SJ, Park SJ, Kwak Y, Kim SY, et al. Impact of multistage cannula on recirculation in various venovenous extracorporeal membrane oxygenation cannulation strategies. ASAIO J. 2025. 10.1097/MAT.0000000000002384. 39963977

[20] Abrams D, Bacchetta M, Brodie D. Recirculation in venovenous extracorporeal membrane oxygenation. ASAIO J. 2015;61(2):115–121. 10.1097/MAT.0000000000000179. 25423117

[21] Wickramarachchi A, Gregory SD, Burrell AJC, Khamooshi M. Flow characterization of Maquet and Bio-Medicus multi-stage drainage cannulae during venoarterial extracorporeal membrane oxygenation. Comput Biol Med. 2024;171:108135. 10.1016/j.compbiomed.2024.108135. 38373368

[22] Bukova M, Schumacher T, Mantl M, Funken D, Hoeffler K, Koeditz H, et al. Factors influencing recirculation in veno-venous extracorporeal membrane oxygenation: insights from a controlled bench study. ASAIO J. 2025. 10.1097/MAT.0000000000002465. 40386968

[23] Gehron J, Bandorski D, Mayer K, Boning A. the impact of recirculation on extracorporeal gas exchange and patient oxygenation during veno-venous extracorporeal membrane oxygenation-results of an observational clinical trial. J Clin Med. 2023;12(2). 10.3390/jcm12020416. PMC9866780 36675344

[24] Antonsen LP, Espinoza A, Halvorsen PS, Schalit I, Bergan H, Lilja D, et al. The impact of hypovolemia and PEEP on recirculation in venovenous ECMO: an experimental porcine model. Intensive Care Med Exp. 2024;12(1):51. 10.1186/s40635-024-00636-5. 38822111 PMC11143165

[25] Togo K, Takewa Y, Katagiri N, Fujii Y, Kishimoto S, Date K, et al. Impact of bypass flow rate and catheter position in veno-venous extracorporeal membrane oxygenation on gas exchange in vivo. J Artif Organs. 2015;18(2):128–135. 10.1007/s10047-014-0810-0. 25477271

[26] Ko UW, Choi CH, Park CH, Lee SI. Alternative position of cannulae in veno-venous extracorporeal membrane oxygenation for maintaining sufficient flow support. Gen Thorac Cardiovasc Surg. 2023;71(6):369–372. 10.1007/s11748-023-01924-9. 36897504

[27] Rich PB, Awad SS, Crotti S, Hirschl RB, Bartlett RH, Schreiner RJ. A prospective comparison of atrio-femoral and femoro-atrial flow in adult venovenous extracorporeal life support. J Thorac Cardiovasc Surg. 1998;116(4):628–632. 10.1016/S0022-5223(98)70170-9. 9766592

[28] Togo K, Takewa Y, Katagiri N, Fujii Y, Yamashita AC, Tastumi E. Optimal drainage cannula position in dual cannulation for veno-venous extracorporeal membrane oxygenation. Int J Artif Organs. 2018;41(12):867–871. 10.1177/0391398818795357. 30223702

[29] Rubino A, Vuylsteke A, Jenkins DP, Fowles JA, Hockings L, Valchanov K. Direct complications of the Avalon bicaval dual-lumen cannula in respiratory extracorporeal membrane oxygenation (ECMO): Single-center experience. Int J Artif Organs. 2014;37(10):741–747. 10.5301/ijao.5000357. 25361182

[30] Lim SY, Ahn S, Hong SB, Chung CR, Jeon K, Lee SM, et al. Clinical outcomes according to cannula configurations in patients with acute respiratory distress syndrome under veno-venous extracorporeal membrane oxygenation: a Korean multicenter study. Ann Intensive Care. 2020;10(1):86. 10.1186/s13613-020-00700-9. 32572593 PMC7306930

[31] Gaies MG, Gurney JG, Yen AH, Napoli ML, Gajarski RJ, Ohye RG, et al. Vasoactive-inotropic score as a predictor of morbidity and mortality in infants after cardiopulmonary bypass. Pediatr Crit Care Med. 2010;11(2):234–238. 10.1097/PCC.0b013e3181b806fc. 19794327

[32] Duffin SC, Askew JH, Southwood TJ, Forrest P, Plunkett B, Totaro RJ. An intensivist-led ECMO accreditation pathway and safety data over the first 4 years. Crit Care Resusc. 2024;26(1):41–46. 10.1016/j.ccrj.2023.11.006. 38690187 PMC11056391

[33] McCanny P, Smith MW, O'Brien SG, Buscher H, Carton EG. Fluid balance and recovery of native lung function in adult patients supported by venovenous extracorporeal membrane oxygenation and continuous renal replacement therapy. ASAIO J. 2019;65(6):614–619. 10.1097/MAT.0000000000000860. 30379653

[34] Song K, Na SJ, Chung CR, Jeon K, Suh GY, Chung S, et al. Optimal position of a femorojugular cannulation for venovenous extracorporeal membrane oxygenation in acute respiratory distress syndrome. Ann Thorac Surg. 2023;115(4):1016–1022. 10.1016/j.athoracsur.2022.10.023. 36967708

[35] Famous KR, Delucchi K, Ware LB, Kangelaris KN, Liu KD, Thompson BT, et al. Acute respiratory distress syndrome subphenotypes respond differently to randomized fluid management strategy. Am J Respir Crit Care Med. 2017;195(3):331–338. 10.1164/rccm.201603-0645OC. 27513822 PMC5328179

[36] Abrams D, Garan AR, Brodie D. Awake and fully mobile patients on cardiac extracorporeal life support. Ann Cardiothorac Surg. 2019;8(1):44–53. 10.21037/acs.2018.08.03. 30854311 PMC6379193

[37] Pooth JS, Forster JK, Benk C, Diel P, Brixius SJ, Maier S, et al. Impact of cannulation strategy and extracorporeal blood flow on recirculation during veno-venous extracorporeal membrane oxygenation. Artif Organs. 2025;49(6):1012–1020. 10.1111/aor.14961. 39868656 PMC12120813

[38] Fisser C, Palmer O, Sallisalmi M, Paulus M, Foltan M, Philipp A, et al. Recirculation in single lumen cannula venovenous extracorporeal membrane oxygenation: A non-randomized bi-centric trial. Front Med (Lausanne). 2022;9:973240. 10.3389/fmed.2022.973240. 36117961 PMC9470851

[39] Conrad SA, Wang D. Evaluation of recirculation during venovenous extracorporeal membrane oxygenation using computational fluid dynamics incorporating fluid-structure interaction. ASAIO J. 2021;67(8):943–953. 10.1097/MAT.0000000000001314. 33315664 PMC8318564

[40] Bonacchi M, Harmelin G, Peris A, Sani G. A novel strategy to improve systemic oxygenation in venovenous extracorporeal membrane oxygenation: the “chi-configuration”. J Thorac Cardiovasc Surg. 2011;142(5):1197–1204. 10.1016/j.jtcvs.2011.01.046. 21397257

[41] Lin TY, Horng FM, Chiu KM, Chu SH, Shieh JS. A simple modification of inflow cannula to reduce recirculation of venovenous extracorporeal membrane oxygenation. J Thorac Cardiovasc Surg. 2009;138(2):503–506. 10.1016/j.jtcvs.2008.02.095. 19619807

[42] Mekontso Dessap A, Boissier F, Charron C, Begot E, Repesse X, Legras A, et al. Acute cor pulmonale during protective ventilation for acute respiratory distress syndrome: prevalence, predictors, and clinical impact. Intensive Care Med. 2016;42(5):862–870. 10.1007/s00134-015-4141-2. 26650055

[43] Heuts S, Ubben JF, Banks-Gonzales V, Sels JW, Lorusso R, van Mook W, et al. Nitric oxide ventilation improves recirculation and right ventricular function during veno-venous extracorporeal membrane oxygenation in a COVID-19 patient. J Cardiothorac Vasc Anesth. 2021;35(9):2763–2767. 10.1053/j.jvca.2020.09.137. 33077329 PMC7534592

[44] van Heijst AF, van der Staak FH, de Haan AF, Liem KD, Festen C, Geven WB, et al. Recirculation in double lumen catheter veno-venous extracorporeal membrane oxygenation measured by an ultrasound dilution technique. ASAIO J. 2001;47(4):372–376. 10.1097/00002480-200107000-00015. 11482489

[45] Lindstrom SJ, Mennen MT, Rosenfeldt FL, Salamonsen RF. Quantifying recirculation in extracorporeal membrane oxygenation: a new technique validated. Int J Artif Organs. 2009;32(12):857–863. 10.1177/039139880903201204. 20037890

[46] Masi P, Gouriet L, Radu C, Folliguet T, Fiore A, Gallet R, et al. Immediate clinical complications occurring during membrane change in patients on veno-venous extracorporeal membrane oxygenation. ASAIO J. 2025;71(2):120–127. 10.1097/MAT.0000000000002270. 39052935 PMC11761025

[47] Walker J, Primmer J, Searles BE, Darling EM. The potential of accurate SvO_2_ monitoring during venovenous extracorporeal membrane oxygenation: an in vitro model using ultrasound dilution. Perfusion. 2007;22(4):239–244. 10.1177/0267659107083656. 18181511

[48] Mazzeffi M, Gutsche J. Thermodilution-derived recirculation and cardiac output measurement during veno-venous extracorporeal membrane oxygenation: Do We need more bells and whistles? Anesthesiology. 2024;140(5):887–889. 10.1097/ALN.0000000000004930. 38592358

[49] Undar A, Wang S, Moroi M, Kunselman AR, Brehm CE. Evaluation and comparison of hemodynamic performance of three ECLS systems in a simulated adult cardiogenic shock model. Artif Organs. 2018;42(8):776–785. 10.1111/aor.13126. 29575097

[50] Joyce PR, Hodgson CL, Bellomo R, Gregory SD, Raman J, Stephens AF, et al. Smaller return cannula in venoarterial extracorporeal membrane oxygenation does not increase hemolysis: a single-center, Cohort Study. ASAIO J. 2023;69(11):1004–1008. 10.1097/MAT.0000000000002027. 37549666

[51] Kelly AM, Weldon D, Tsang AY, Graham CA. Comparison between two methods for estimating pneumothorax size from chest X-rays. Respir Med. 2006;100(8):1356–1359. 10.1016/j.rmed.2005.11.022. 16406560

