# Supplementary material for: Cannula tip separation and configuration as determinants of recirculation in venovenous ECMO
Source: J Extra Corpor Technol. 2026 Sep 15;58(3):255–66. doi: 10.1051/ject/2026010 (PMC13577619; doi:10.1051/ject/2026010)
Supplement: Supplementary file 1 [file ject-58-255-s1.pdf]

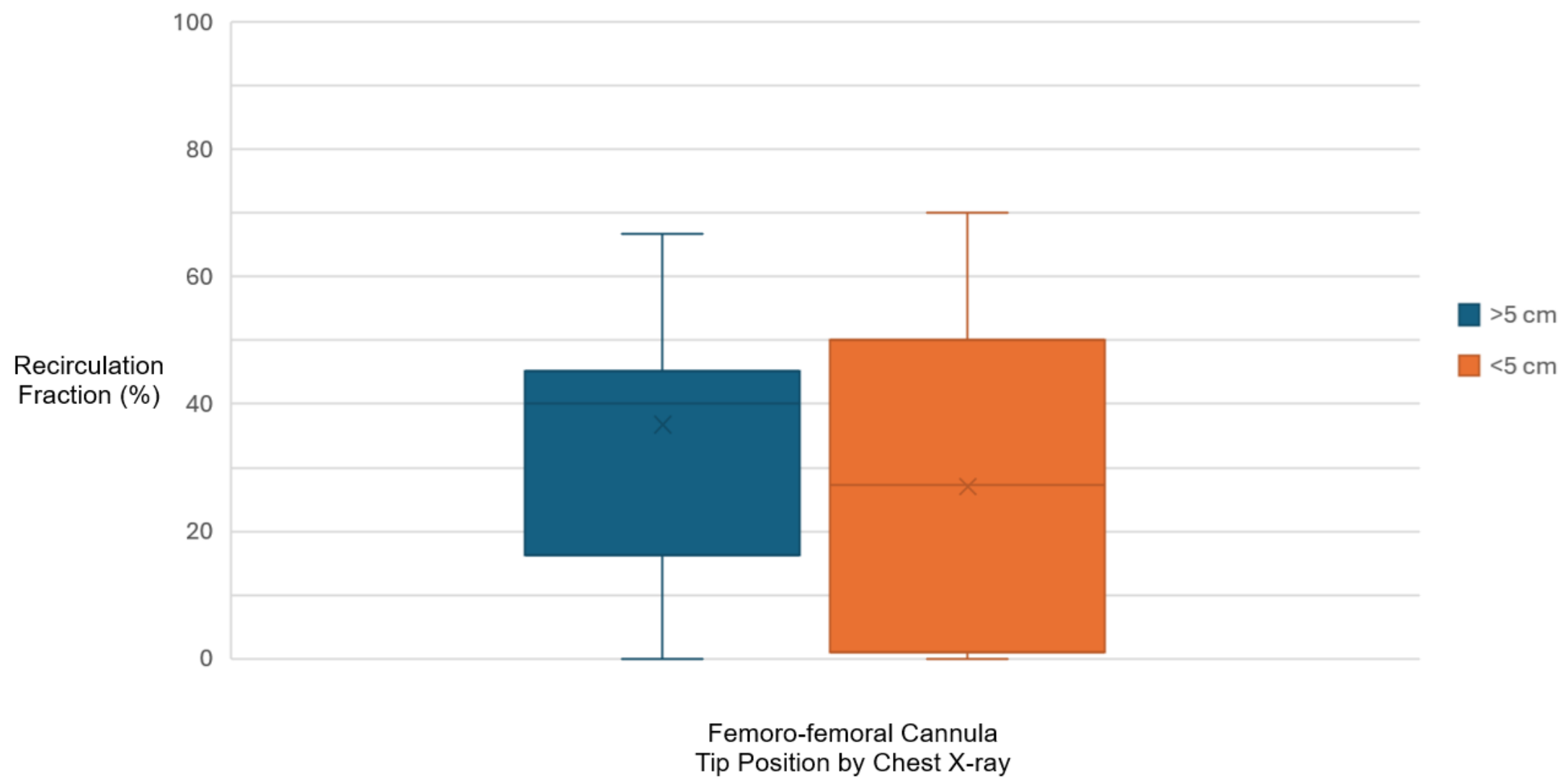

Supplementary Figure 1.

Box-and-whisker plot showing recirculation fraction (%) in patients with femoro-femoral venovenous ECMO, stratified by drainage-to-return cannula tip separation on chest radiograph. Patients are grouped by separation distance <5 cm or ≥5 cm as assessed by an experienced ECMO specialist.

No significant difference in recirculation fraction was observed between groups.

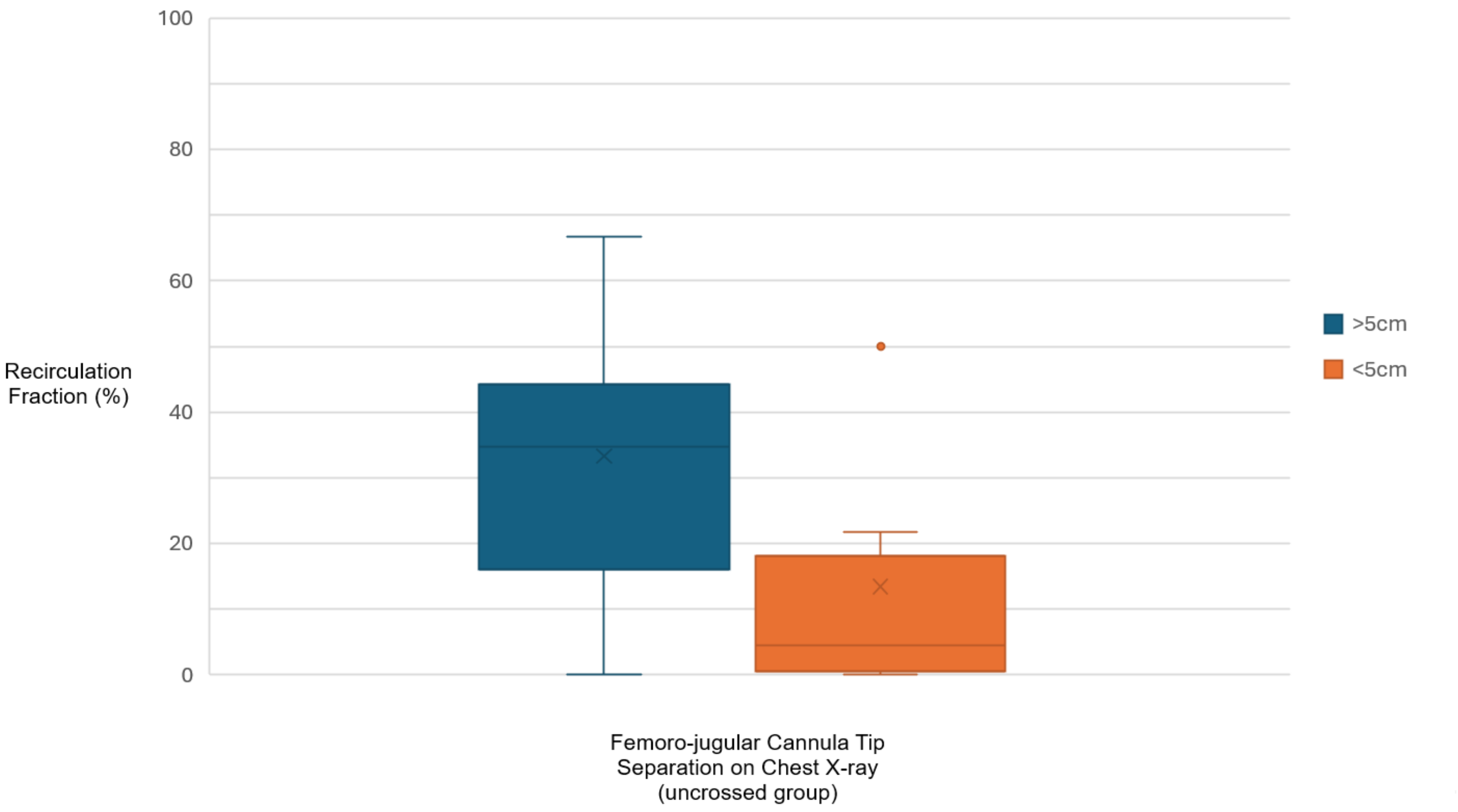
100
80
60
40
20
0
Recirculation Fraction (%)
>5cm
<5cm
Femoro-jugular Cannula Tip Separation on Chest X-ray (uncrossed group)

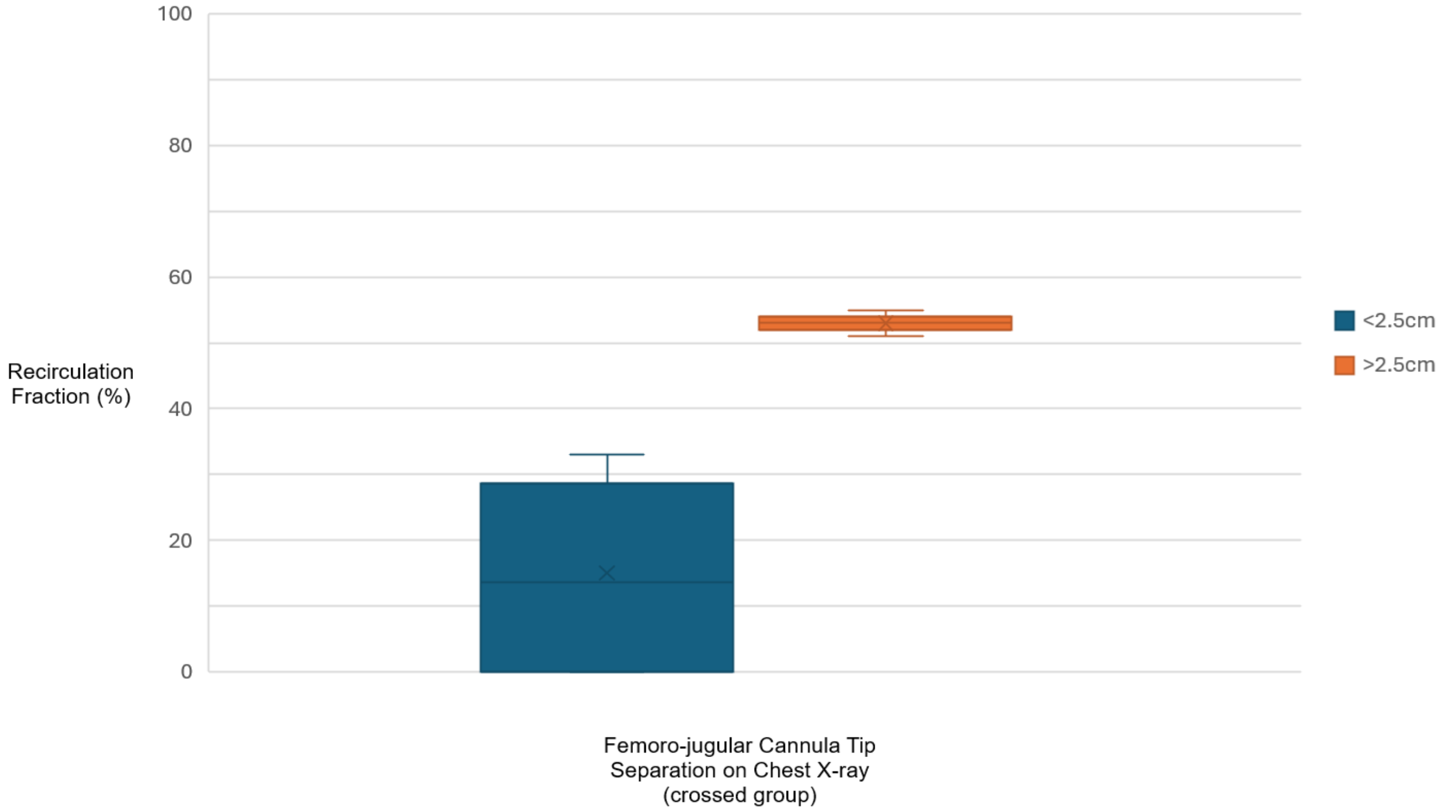
100
80
60
40
20
0
Recirculation Fraction (%)
<2.5cm
>2.5cm
Femoro-jugular Cannula Tip Separation on Chest X-ray (crossed group)

Supplementary Figure 2.

Box-and-whisker plots showing recirculation fraction (%) in femoro-jugular venovenous ECMO patients, stratified by cannula tip separation on chest radiograph.

Top panel: Patients with uncrossed cannulas, grouped by separation <5 cm vs ≥5 cm. No statistically significant difference.

Bottom panel: Patients with crossed cannulas, grouped by separation <2.5 cm vs ≥2.5 cm. Recirculation fraction was significantly lower in patients with greater tip separation ($p < 0.01$).

	All	FF	FJ	P value	Cross	Uncrossed	P value^	p value

Supplementary Table 1. Assessment of potential recirculation in 99 patients excluded from the primary study based on SvO_2 monitoring:

Here SvO2 >75% was considered significant, if >4 hours or associated with a patient or ECMO intervention e.g cannula movement this was declared 'prolonged'. Hypoxaemia potentially related to recirculation was defined as SpO2 <88% at any time the SvO2 was greater than 75%.

The EMR was examined for patient demography and for initial cannula configuration (FF/FJ – crossed/uncrossed), and for record of patient or ECMO interventions to combat recirculation or hypoxaemia related to recirculation. This data is presented below.

There were more males in the FJ uncrossed configuration than other configurations. The median access cannula for FJ crossed tended to be larger (29Fr). The incidence of a raised SvO2 at any time was 50.5% in the overall cohort and not statistically significantly different between configurations. The rate of sustained high SvO2 was 32.3%. Hypoxaemia was documented in 7.1% of patients overall in this sub cohort, and sustained hypoxaemia occurred in 5.1% of patients. Only ten patients underwent interventions specifically to address recirculation or recirculation related hypoxaemia. This was predominantly manipulation of cannula position, eg distraction of the access and return cannula in the uncrossed Fj configuration. One patient underwent upgrade to high flow VV ECM. ECMO survival was higher in uncrossed vs crossed FJ configuration but not significant between all groups. Grossly, patients in this sub cohort did not appear different to those included in the formal study.

								*
**Number**	**99**	17 (17.2%)	82 (82.8%)		30 (36.7%)	52 (63.3%)		
**Sex (male)**	60.0%	58.8%	63.4%	0.63	40.0%	76.9.%	**0.0008**	**0.01**
**age**	46.0 (33;56.5)	52.0 (29;59)	44.5 (33;54.75)	0.5	44.5 (32.5;53. 75)	44.5 (34.5;55)	0.38	0.7
**Median access cannula (Fr)**	25	25	25		29	25		
**Median return cannula (Fr)**	21	21	21		21	21		
**SvO2 high (any)**	50 (50.5%)	8 (47.1%)	42 (51.2%)	0.8	14 (46.7%)	28 (53.8%)	0.64	0.83
**SvO2 high (sustained)**	32 (32.3)	8 (47.1%)	24 (29.3%)	0.17	8 (26.7%)	16 (30.8%)	0.80	0.49
**Hypoxaemia (SpO2<88%)**	7 (7.1%)	2 (11.8%)	5 (6.1%)	0.35	1 (3.3%)	4 (7.7%)	0.65	0.71
**Sustained Hypoxaemia**	5 (5.1%)	1 (5.9%)	4 (4.9%)	1	1 (3.3%)	3 (5.8%)	1.00	0.97
**Remedial Interventions (documented)**	10 (10.1%)	1 (5.9%)	9 (10.9%)	1	2 (6.6%)	7 (13.5%)	0.47	0.71
		1 cannula manipulation			2 cannula manipulations	6 cannula manipulations<br>1 high flow conversion		
**ECMO duration (days)**	8 (4.75;13.25)	8 (3.75;70.6)	8 (5;17.3)	0.45	7 (5;13)	9 (5.5;17.5)	0.39	0.31
**ECMO survival (%)**	70.7%	70.6%	70.7%	1.0	63.3%	75.0%	**0.01**	0.74
**Mechanical ventilation duration (days)**	11 (10;31.25)	9 (7;17)	21 (8;31)	0.33	19 (6;29.5)	21 (10.5;33.5)	0.34	0.12
**Tracheostomy (%)**	25.3%	29.4%	24.4%	0.76	13.3%	30.8%	0.11	0.35
**ICU survival (%)**	53.5%	52.9%	53.7%	1.00	43.3%	63.5%	0.17	0.57

Fishers exact test or Chi2 test for categorical variables, Mann Whitney U test or one way ANOVA for continuous variables

^p values for FJ crossed Vs FJ uncrossed

*p values for 3 group comparison

Significant results, more Males in the uncrossed vs crossed FJ configuration

Higher ECMO survival in the uncrossed vs crossed FJ configuration

No difference in ICU mortality between the FF subgroup and whole cohort nor FJ whole and sub

Supplementary Table 2. Cannula sizes used by configuration

**Variable**	**FJ (n = 17)**	**FF (n = 7)**
**Drainage Cannula (Fr)**		
21	1 (5.8%)	–
25	7 (41.2%)	5 (71.4%)
29	9 (52.9%)	2 (28.6%)
**Return Cannula (Fr)**		
19	6 (35.3%)	–
21	5 (29.4%)	7 (100%)
23	6 (35.3%)	–

Legend

Frequency and proportion of drainage and return cannula sizes used in patients with femoro-jugular (FJ) and femoro-femoral (FF) venovenous ECMO configurations. Data are presented as number (% of group). Dashes indicate that no patients in that group received that cannula size.

Key

FJ: Femoro-jugular configuration; FF: Femoro-femoral configuration; Fr: French (catheter size)

Supplementary Table 3: Recirculation by Drainage and Return Cannula Size in FJ and FF Configurations

**Drainage Cannula (Fr)**	**FJ n (%)**	**FJ Rf (%)**	**FF n (%)**	**FF Rf (%)**
**21**	**2 (7.7%)**	**56.5 (34.8–78.3)**	**–**	**–**
**25**	**8 (30.8%)**	**27.3 (9.9–47.5)**	**5 (71.4%)**	**–**
**29**	**16 (61.5%)**	**31.2 (18.0–44.1)**	**2 (28.6%)**	**–**
All 25Fr Access Rf: **41.5 (11.4–50.0) vs** All 29Fr Access Rf: **29.4 (13.9–40.5), ns (p = 0.47)**				
**Return Cannula (Fr)**	**FJ n (%)**	**FJ Rf (%)**	**FF n (%)**	**FF Rf (%)**
**19**	**2 (7.7%)**	**31 (19–43)**	**0 (0%)**	**–**
**21**	**11 (42.3%)**	**20.2 (0–52.5)**	**7 (100%)**	**41.5 (38.3–45.2)**
**23**	**13 (50%)**	**33 (25–42.1)**	**0 (0%)**	**–**
All 21Fr Return Rf: **38.3 (6.7–50.0) vs** All 23Fr Return Rf: **33.0 (25.0–42.1), ns**				

Legend

Recirculation fraction (Rf%) shown according to drainage and return cannula size for patients supported with femoro-jugular (FJ) and femoro-femoral (FF) venovenous ECMO configurations. Data are presented as number (% of group) and median Rf% with interquartile range (IQR). Comparisons of Rf between sizes are shown for common cannula pairings. No statistically significant differences were observed between groups. Dashes indicate that no patients in that group received that cannula size.

Key

FJ: Femoro-jugular configuration; FF: Femoro-femoral configuration; Fr: French (catheter size); Rf: Recirculation fraction; IQR: Interquartile range; ns: Not significant
